# Evolution of emergency medical services in the Kingdom of Bahrain

**DOI:** 10.1186/s12245-020-00280-2

**Published:** 2020-04-28

**Authors:** Feras H. Abuzeyad, Ghada Al Qasim, Leena Alqasem, Mudhaffar I. Al Farras

**Affiliations:** 1 Department of Emergency Medicine, King Hamad University Hospital, Building 2345, Road 2835, Block 228, P. O. Box 24343, Busaiteen, Kingdom of Bahrain; 2 Emergency Medicine Department, Bahrain Defence Force, Royal Medical Services, Riffa, Kingdom of Bahrain; 3 National Health Regulatory Authority, Sanabis, Kingdom of Bahrain

**Keywords:** Emergency medical services, Kingdom of Bahrain, Evolution, Prehospital care

## Abstract

Emergency medical services (EMS) is crucial to any healthcare system, especially in urban countries. The Kingdom of Bahrain has always strived to develop healthcare services throughout the Kingdom including EMS. Like any other country, the Kingdom has gone through several stages in the provision of EMS. This article will focus on the development of EMS in the Kingdom and its evolution from a scattered hospital-based system to a unified system, which ensures ease of access for the population and speed of delivery to the healthcare facilities. The major focus will be the most recent national project which is the National Ambulance.

## Background

**Fig. 1:**
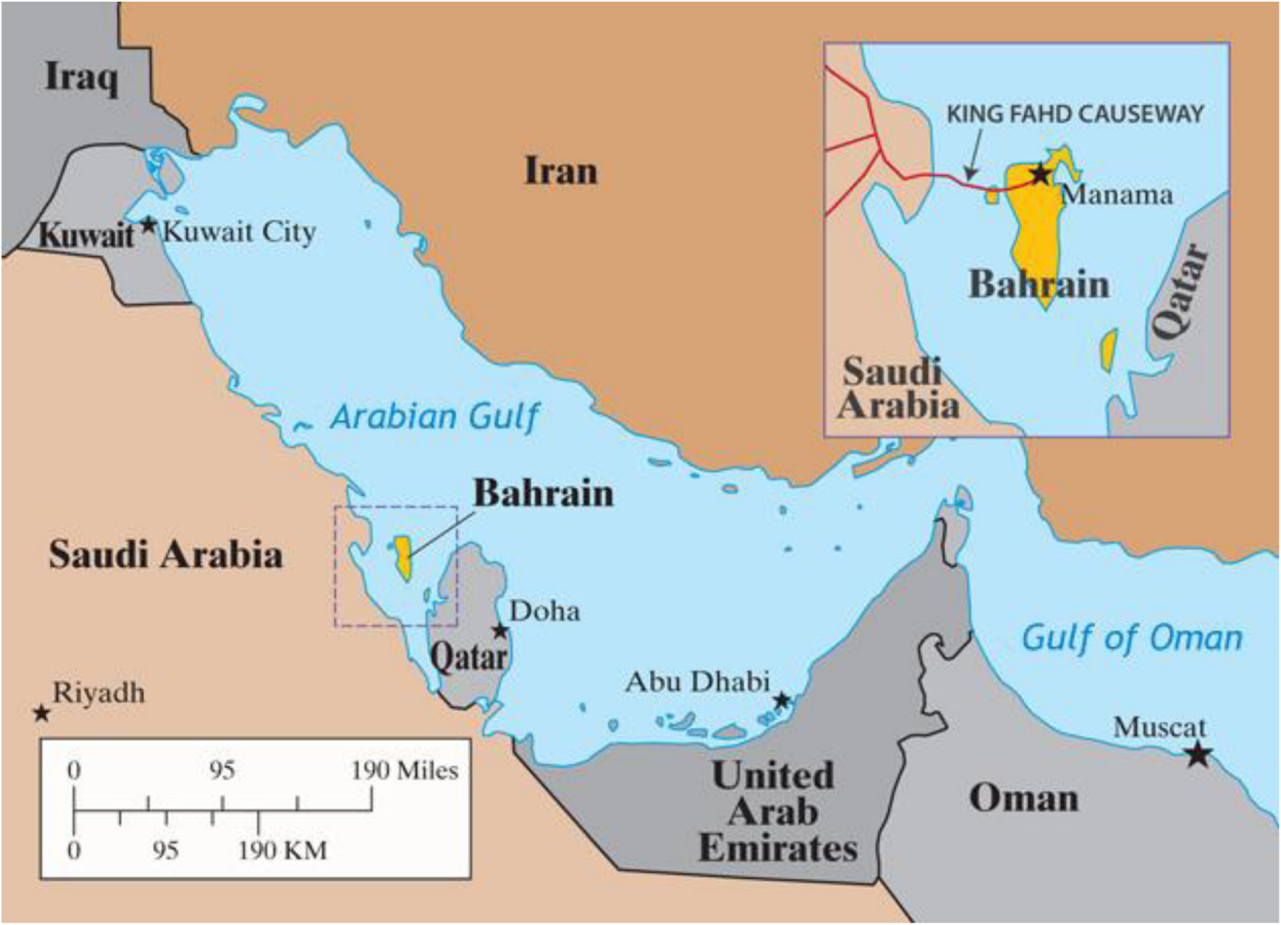
Kingdom of Bahrain Map

The Kingdom of Bahrain is an archipelago of 33 islands in the Arabian Gulf to the east coast of Saudi Arabia and the north-west coast of the State of Qatar, and it is linked to Saudi Arabia by King Fahd Causeway [1] (Fig. 1). The country has a land area of 770 km^2^, which is occupied by an approximate population of 1.6 million [2]. The Kingdom is divided into four governances: the Capital, Muharraq, Northern, and Southern and has a population density of 1761 people/Km^2^ [3]. In 1971, the country achieved independence and became a member of the United Nations. In 1981, Bahrain became a member of the formulated Gulf Cooperation Council (GCC) [2].

## Health care overview in the Kingdom of Bahrain

The healthcare system in Bahrain evolved in the early twentieth century, and similar to other GCC countries, oil and natural gas production revenues helped to establish the healthcare systems, with an estimate of 5% of the country’s gross domestic product (GDP) being spent on healthcare [4]. The Kingdom is covered by a total of 24 governmental and private hospitals, where primary healthcare services are provided through 28 public health centers [3]. There are a total of 14.9 physicians per 10,000 population, which ranks the Kingdom as the fifth among the six GCC countries in terms of physician numbers. Additionally, the Kingdom has 18 hospital beds for the same number of the population [5].

## Overview of emergency medical services (EMS)

The World Health Organization (WHO) strongly supports the integration of EMS as an essential component of the healthcare system due to the increased burden of diseases, especially trauma and cardiovascular diseases, and the right for individuals to get access to healthcare services including EMS [6]. EMS as a subspecialty was recognized by the American College of Emergency Physicians (ACEP) and the Australian Medical Council [7]. EMS is defined as a comprehensive system that provides the arrangements of personnel, facilities, and equipment for the effective, coordinated, and timely delivery of health and safety services to victims of sudden illness or injury [8]. Worldwide models of EMS systems range from an informal primitive model to a formal complete structure, with the objectives of early incident notification, dispatching proper resources to the scene, giving immediate adequate care, and patient transport to the right healthcare institution [9]. VanRooyen et al. proposed five EMS models commonly used which include hospital-based system, jurisdiction-directed system, private system, volunteer system, and complex system [10]. On the other hand, EMS staffing only features two models: the Anglo-American model which is based on non-physicians and the Franco-German model which is based on physicians [9, 11]. EMS plays a significant role in the management of some prehospital conditions that include cardiac arrest, trauma, acute myocardial infarction (AMI), stroke, sepsis, and behavioral emergencies to reduce the morbidity and mortality outcome of those diseases [11], moreover, shortening the response time [12]. EMS has evolved to be an important integrated part of any healthcare system [9, 10] and in the UK, and it is estimated that the annual requests for EMS are increasing by 8% [13], a figure that can vary globally.

### History of EMS in Bahrain

**Fig. 2:**
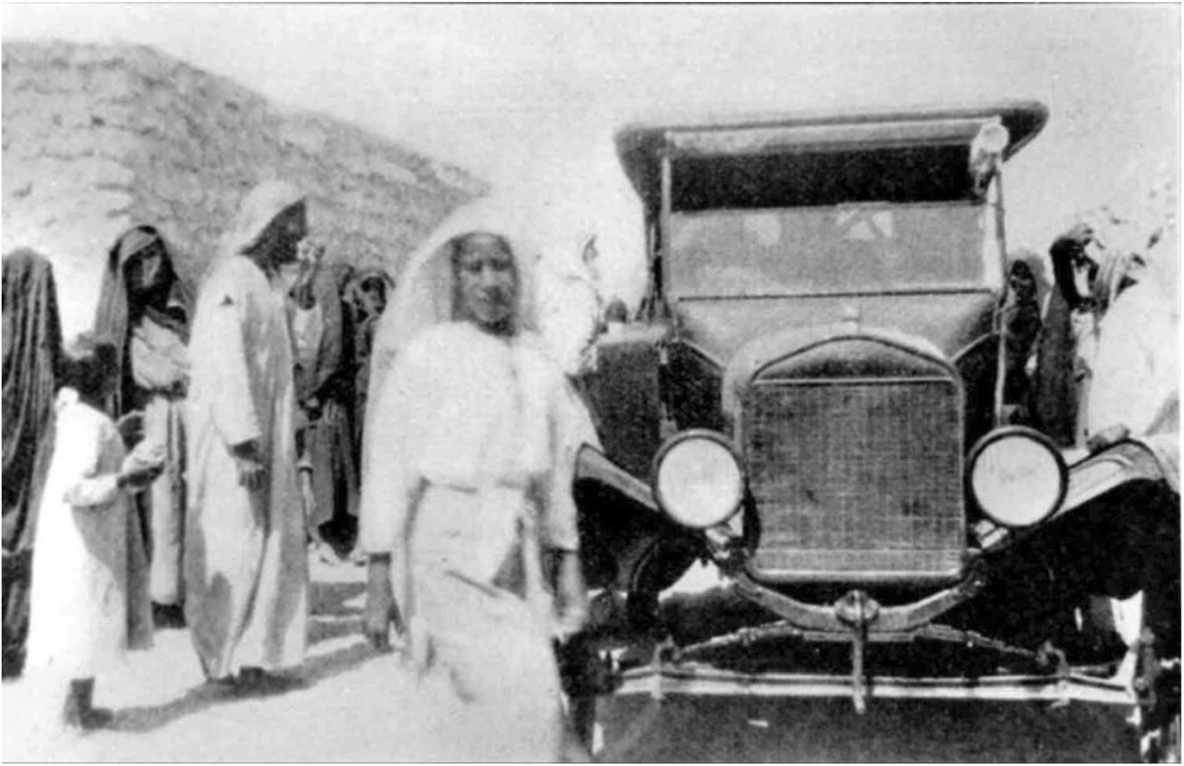
“The Little Traveler” is the model T Ford used by the mission in the early twentieth century

In the early 1900s, the Reform Church of America built the American Mission Hospital formerly known as the Mason Memorial Hospital which was the first hospital to be built on the main island. The hospital provided a vehicle to transport a physician to patients who could not reach the hospital [14], and the vehicle was called “The Little Traveler” in 1920 (Fig. 2) [15]. In 1937, the Bahrain Petroleum Company (BaPCO) established a hospital that provided ambulance transportation services only [14].

In 1950, the Ministry of Health (MOH) started its ambulance transportation services through a donated ambulance from the British army, and the first hospital-based EMS system was established in Salmaniya Medical Complex in 1985 [4, 14], followed by Bahrain Defense Force Hospital in 1995 and King Hamad University Hospital in 2011, as each hospital covers certain geographic areas. In 2007, the WHO approved Bahrain‘s formal EMS system with the emergency number “999” as the national universal access number [4].

Due to urbanization, expanded healthcare, and increased economic growth, the EMS system needed to adapt and expand [10], which led to the proposal of a National Ambulance (NA) in 2011 to unify and centralize the EMS in the Kingdom [4].

On 15 June 2019, the unified vision was achieved, and the official launch of the National Ambulance under the Ministry of Interior (MOI) was announced, which includes the main NA control center and 13 dispatch stations [16]. The EMS time event history in the Kingdom is summarized in Table 1.

### The National Ambulance (NA) and the current EMS system

The call to establish a modern EMS system in the kingdom goes back to 1979 in the article “Emergency Medical Service in Bahrain – The time is now” written by a senior Bahraini orthopedic surgeon at that time, and it took 40 years to achieve this vision in the Kingdom [17]. Establishing and maintaining any EMS system need significant resources [9]; MacFarlane and Benn stated that there is no uniform EMS system that can necessarily fit all countries and suggested some valid points that must be considered in the EMS setting [18]. There are 15 essential components to be considered in the assessment and establishment of any basic EMS system [10]. This led to the formation of the NA committee by MOI in mid-2011, which included among the other three qualified emergency physicians, one representing each of the three main hospitals. The committee was given the following responsibilities: (1) assess the current ambulance services in the Kingdom and map out the shortcomings and existing difficulties, (2) improve the current system through a unified control center and dispatch stations, (3) identify the necessary technical, organizational, manpower, and financial needs, and (4) develop stages of operation of the project. The committee started with analyzing the current EMS system, its components, different stakeholders, and the existing resources. The committee later began the implementation process by listing all its needs and finally proposed the project in stepwise phases, which included (1) estimating the number of ambulances required and the number of paramedics and ambulance nurses needed, (2) setting up detailed lists of required equipment for the ambulances, control center, and dispatch stations, (3) visited Abu Dhabi National Ambulance to look at their EMS systems and learn from their experiences, [19] (4) setting up the organizational chart for the NA, (5) interviewing potential paramedics and ambulance nurses for recruitment, and (6) inspecting the equipment, ambulances, control center, and dispatch stations.

The committee also considered the short prehospital response time in the country and having receiving hospitals with well-structured emergency departments and trauma services that was an advantageous scenario [18]. Some of the challenges faced by the committee included delays in the construction of the NA control center and dispatch stations, purchasing the ambulances and equipment, recruiting and training paramedics and ambulance nurses, recruiting quality control staff, formulating the policies, assurance of continuity of availability of supplies for the ambulances to ensure continuity in the provision of services, delays in the establishment of MPDS, and most importantly, allocating a yearly financial budget by the government. In 2019, the NA was officially in operation [16], and that represents a real transformation in EMS from a fragmented hospital-based service to a unified central service that provides a formal EMS system to the whole kingdom. The result was a change from a hospital-based model to a third-service model (under government/state) [20] that follows the Anglo-American model.

### The control center and the dispatch stations

**Fig. 3:**
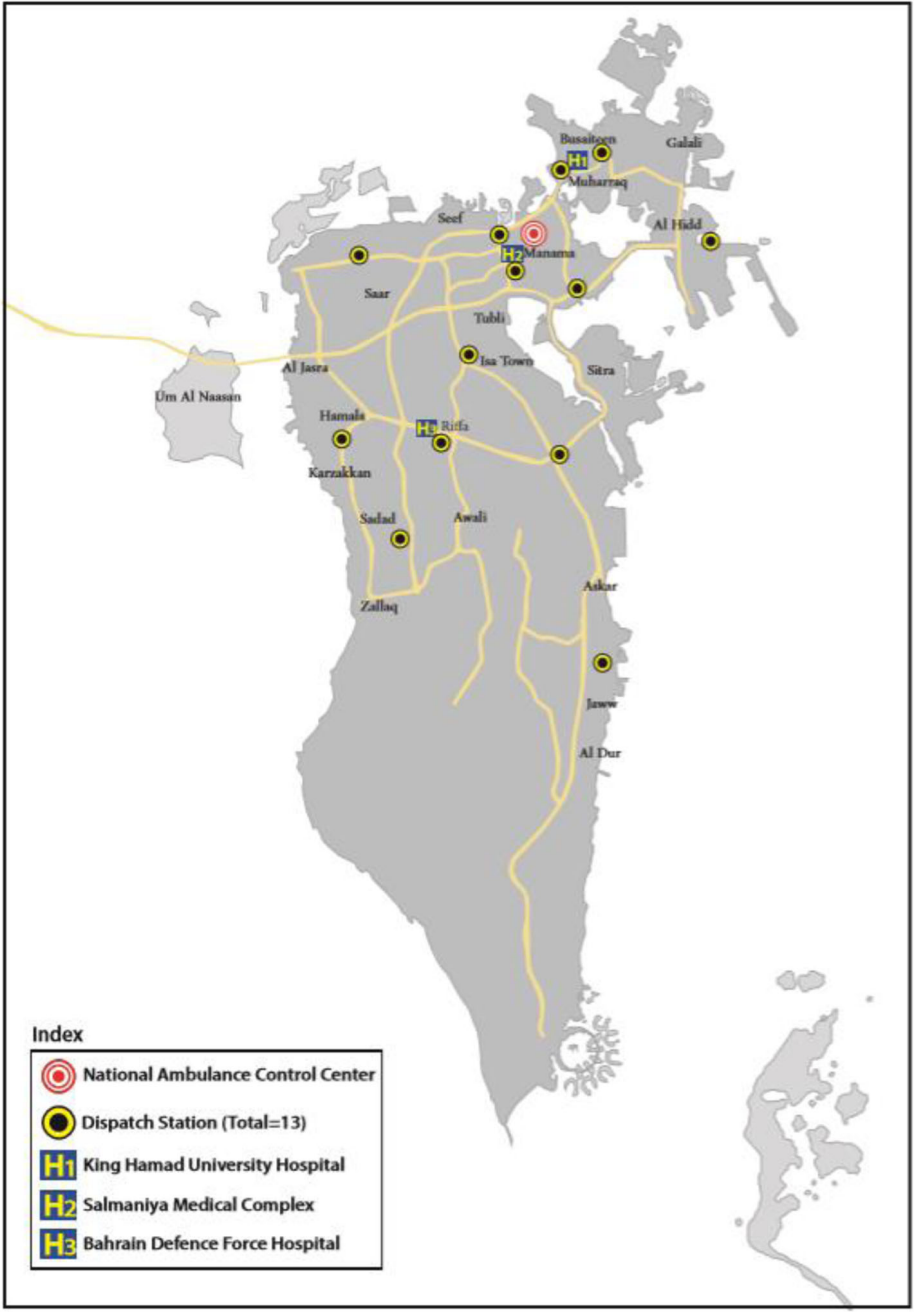
The National Ambulance control center and its 13 dispatch stations in the Kingdom of Bahrain

The main NA control center is under the MOI, and it controls 13 dispatch stations that cover the whole country (Fig. 3). The center hosts the call takers who receive all “999” calls and use the Medical Priority Dispatch System (MPDS) to evaluate different medical complaints and dispatch the EMS teams accordingly [21]. However, looking at different dispatch systems there is weak evidence for their accuracy. The center uses two-way communications through radios and phones to exchange patient’s information, with emergency physicians available to provide the online medical direction to the prehospital care providers. The ambulance vehicles are linked with a global position system (GPS), with a moving digital map in the center, which helps to track, guide, and speed arrival to the scene [22].

**Fig. 4:**
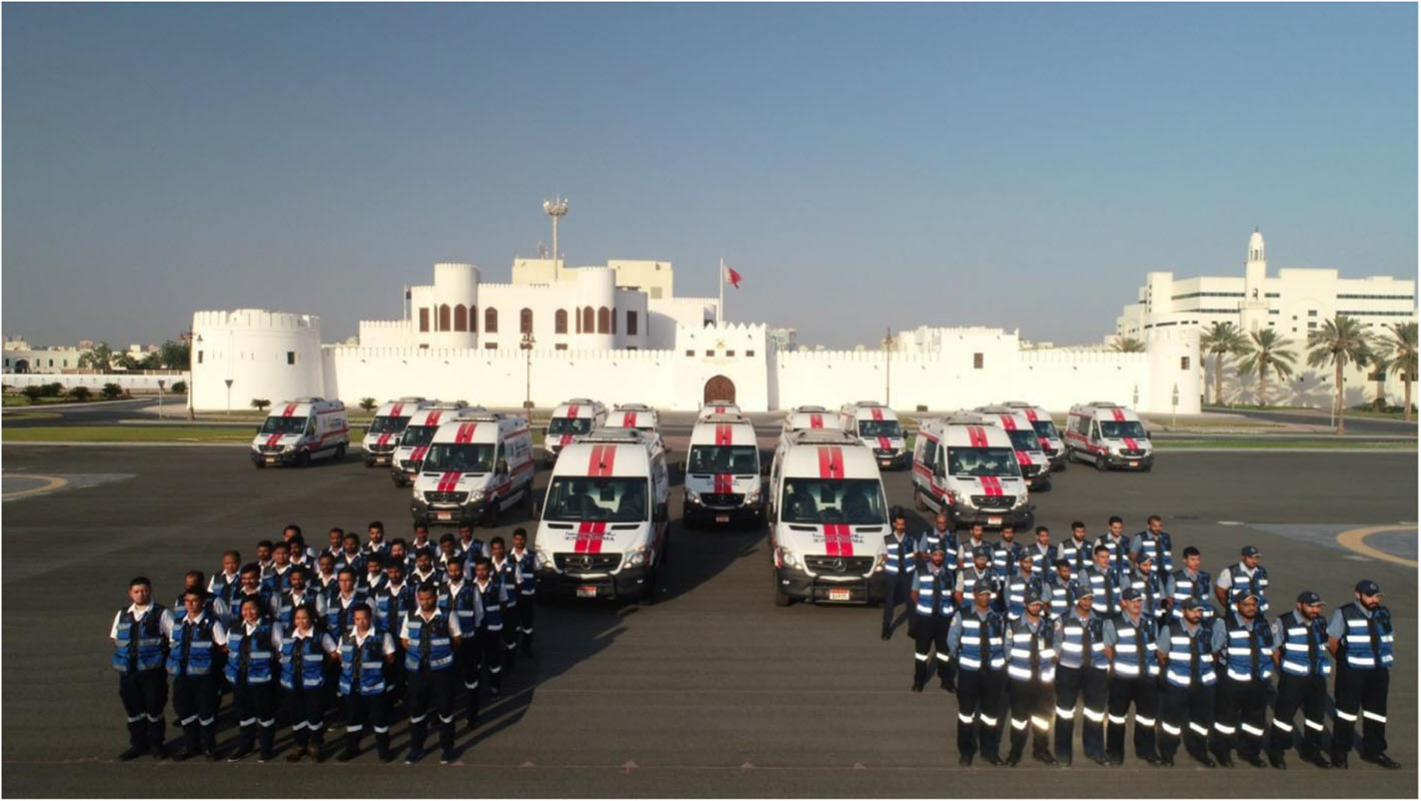
National Ambulance ground ambulance fleet

NA has built 10 identical dispatch stations in addition to the 3 existing dispatch stations in the 3 main hospitals (SMC, BDFH, and KHUH) to have a total of 13 stations. The NA has a fleet of 36 ambulance vehicles type II (Mercedes Sprinter) (Fig. 4) and each dispatch station hosts 2 ambulance vehicles.

#### Personnel

Ambulance staffing follows the Anlo-American model with paramedics (Bachelor’s degree) or ambulance nurses, which is similar to regional GCC countries [9, 23–25]. The staff follows pre-written clinical protocols and is supported by online medical directions if needed. All prehospital care providers must be licensed by the National Health Regulatory Authority (NHRA). In addition, they need to maintain the validity of the following courses: basic life support (BLS), advanced cardiac life support (ACLS), pediatric advanced life support (PALS), and prehospital trauma life support (PHTLS) [26], to obtain and renew their professional licenses to practice.

#### Type of service and equipment

Despite the controversies in providing BLS versus Advanced Life Support (ALS) in prehospital care [27], the NA decided to provide a single tier uniform ALS services to all patients and supplied its ambulances with BLS and ALS equipment. This uniform EMS response model can be implemented easily, as it ensures the availability of ALS providers at the scene and is cost-effective [13].

#### Helicopter emergency medical services (HEMS)

Multiple studies have shown that when compared to ground ambulance transportation, HEMS did not speed up hospital arrival, had no survival or mortality benefits, compromised patient safety, and was a costly service [28]. The NA provides HEMS services only in rare situations where critical patients must be transferred from ships in the sea or from isolated islands to the hospital. The two dedicated helicopters are Bell 412 type and are supplied with the needed trained staff and equipment.

#### National Ambulance benefits

Converting the fragmented EMS system with multiple providers into a unified organization has improved and standardized the prehospital services provided, enabled appropriate data collection and analysis, and enhanced the communication between the relevant parties. NA also has a target of public education and patient satisfaction assessment which are assigned to the recently developed quality department. Feedback from the transferred patients on their satisfaction levels is obtained through phone calls and short text message via mobile phones. The NA’s success resulted from preexisting conditions in the Kingdom including its small geographic area, urbanization, good infrastructure, and the increase in the number of dispatch stations [2, 4], with a vision to shorten the response time in trauma and other emergency conditions to speed up the hospital arrival times by using a uniform ALS response system [13].

The EMS system was developed initially to reduce the morbidity and mortality from trauma and cardiac cases [29], and these two conditions benefited the most from the NA. The death rate per 100,000 from motor vehicle accidents in Bahrain is 15.74, and though this is a high figure, it is comparably lower than other GCC countries [30]. Gulf RACE-2Ps is an important study conducted in the GCC countries on EMS use by patients who presented with ST-elevation AMI that showed significant underutilization of the services provided. In that same study, only 40.7% of such patients were managed and transported by EMS in Bahrain [31]. The development of NA with organized ALS care will benefit patients with road traffic injuries and reduce the burden of trauma on the country [32], as well as cardiac patients.

### Service d’Aide Médicale d’Urgence (SAMU)

When comparing the Anglo-American model (where the patient is delivered to the physician) vs. the Franco-German model (where the physician is delivered to the patient) [33], there is no evidence from the literature to support one model over the other, and the selection should be based on the public’s needs and the available resources [26]. In 2011, this opened a debate among officials in the Kingdom on which model to be used, and a French team from the Service d’Aide Médicale d’Urgence (SAMU) was invited to Bahrain to explore their model and experiences. However, the NA committee analysis on the current EMS system gave the advice to continue utilizing and improving the current Anglo-American EMS-based system. The decision was for the following reasons: (1) the country’s three main hospitals are also following the Anglo-American system, and there was great resistance to change their operating structure to suit the Franco-German system. (2) It is easier and more cost-effective to work with the existing system and expand it. (3) Introducing a large number of emergency physicians into the EMS system will carry expenses that cannot be maintained. Later, an agreement was signed with the SAMU team to act as an independent advisory body to evaluate and provide advice for the project during and after implementation.

#### Challenges

The NA faces many challenges similar to other EMS agencies. Although the development of NA had benefited, resolved, and facilitated the prehospital care provided to the community, some challenges remain to be solved.

There is an increased demand for EMS services by low acuity patients which increases the load on the emergency department (ED) in the receiving hospitals [34], and 30–50% of all EMS transfers do not qualify to be transferred to the ED [13]. This might be due to the inaccurate MPDS used [22], and the free EMS services provided in Bahrain [4]. The issue will need public education and prevention programs.

The majority of the recruited prehospital care providers are ambulance nurses, with few paramedics. To reverse the situation, Bahraini nationals need to be trained in the field of paramedics in the long-term. Bahrain does not have an EMS college despite the existence of other healthcare allied programs in the country. Fortunately, close neighbors like the Kingdom of Saudi Arabia have a good number of existing governmental and private EMS colleges offering a bachelor’s degree [35].

The evidence-based practice in EMS faces many barriers that must be overcome and lacks the motivation to be implemented [36]. Bahrain is at par with other countries in terms of neglecting EMS research, which creates a gap in healthcare practice [37]. There is a deficit in key performance indicator (KPI) publications. In Asia, for example, only 9 studies looked at the response time as a KPI in the evaluation of EMS services, despite the emphasis by the WHO to achieve an optimal response time of 8 min or less [38].

This takes us to the final challenge, developing and measuring the quality of EMS services provided and establishing KPIs for the NA that match the international recommendations. To achieve this, we must realize that the EMS system is a complex service that lacks data and KPI analysis when looking into its structure-process-outcome system. The EMS system must be evaluated separately from the ED [33]. EMS agencies are advised on the importance of monitoring the relevant and necessary KPIs. This ensures that their services possess the standard quality and safety measures it needs, and at the same time, considers the difficulty in comparing and benchmarking its data with other institutions due to the variation in EMS systems and the selected KPIs [39]. Initially, NA will focus on the response time as the main KPI, where the average current response time in the Kingdom is 11.49 min [14]. This will be combined with patients’ outcomes in trauma and cardiac cases. Also, the existing NA MPDS allows for generating data from the received calls through its own quality case review software. At this stage, it is still too early to release quality statistics from the NA.

## Conclusion

The EMS system in the Kingdom of Bahrain was founded earlier compared to neighboring GCC countries, and the birth of the NA in the Kingdom represents a complete formal universal EMS system that was customized to meet the growing urbanization, increased economic demands, and advancement of the healthcare system. The Anglo-American model was chosen as it better fits the country’s system. As of now, the NA is integrated with the existing healthcare facilities to elevate the quality of the prehospital services provided. Despite this great achievement, there are still many challenges and improvements that need to be resolved and achieved soon. The project represents a single country experience, and it is worth sharing for the potential benefits of others.

## Data Availability

Not applicable.
