# Correction to: Evolution of emergency medical services in the Kingdom of Bahrain

**DOI:** 10.1186/s12245-020-00315-8

**Published:** 2020-11-23

**Authors:** Feras H. Abuzeyad, Ghada Al Qasim, Leena Alqasem, Mudhaffar I. Al Farras

**Affiliations:** 1 Department of Emergency Medicine, King Hamad University Hospital, Building 2345, Road 2835, Block 228, P. O. Box 24343, Busaiteen, Kingdom of Bahrain; 2 Emergency Medicine Department, Bahrain Defence Force, Royal Medical Services, Riffa, Kingdom of Bahrain; 3 National Health Regulatory Authority, Sanabis, Kingdom of Bahrain

**Correction to: Int J Emerg Med 13, 20 (2020)**

**https://doi.org/10.1186/s12245-020-00280-2**

Following publication of the original article [1], it was reported that Table 1 was missing from the published article. Table 1 is provided in this Corrected article and the original article has been corrected.

**Table 1:** EMS Time-event History in the Kingdom of Bahrain

Year	Institution	Type of EMS Provided
1920	American Mission Hospital	Ambulance transportation for a physician to reach patients in the rural area
1937	Bahrain Petroleum Company (BaPCo)	Ambulance transportation to hospital
1950	Ministry of Health	Ambulance transportation to the hospital
1985	Salmaniya Medical Complex (SMC)	Hospital-based EMS system
1995	Bahrain Defense Force Hospital	Hospital-based EMS system
2007	Ministry of Health	WHO recognition of the Kingdom EMS system with ‘999’ as the national emergency access number
2011	King Hamad University Hospital (KHUH)	Hospital-based EMS system
2019	National Ambulance (NA)	Unified, central, and formal EMS system for the Kingdom
